# Role of miR-148a in Hepatitis B Associated Hepatocellular Carcinoma

**DOI:** 10.1371/journal.pone.0035331

**Published:** 2012-04-09

**Authors:** Ke Yuan, Zhaorui Lian, Bill Sun, Marcia M. Clayton, Irene O. L. Ng, Mark A. Feitelson

**Affiliations:** 1 Department of Biology, College of Science and Technology, Temple University, Philadelphia, Pennsylvania, United States of America; 2 Center for Biotechnology, Sbarro Health Research Organization, College of Science and Technology, Temple University, Philadelphia, Pennsylvania, United States of America; 3 Department of Pathology, University of Hong Kong, Queen Mary Hospital, Pokfulam, Hong Kong; Drexel University College of Medicine, United States of America

## Abstract

Hepatitis B virus encoded X antigen (HBx) is a *trans*-regulatory protein that alters the activity of selected transcription factors and cytoplasmic signal transduction pathways. HBx transcriptionally up-regulates the expression of a unique gene, URG11, which in turn transcriptionally up-regulates β-catenin, thereby contributing importantly to hepatocarcinogenesis. HBx and URG11 also alter the expression of multiple microRNAs, and by miRNA array analysis, both were shown to promote the expression of miR-148a. Elevated miR-148a was also seen in HBx positive liver samples from infected patients. To study the function of miR-148a, anti-148a was introduced into HepG2 and Hep3B cells stably expressing HBx or stably over-expressing URG11. Anti-miR-148a suppressed cell proliferation, cell cycle progression, cell migration, anchorage independent growth in soft agar and subcutaneous tumor formation in SCID mice. Introduction of anti-miR-148a increased PTEN protein and mRNA expression, suggesting that PTEN was targeted by miR-148a. Anti-miR-148a failed to suppress PTEN expression when co-transfected with reporter gene mutants in the 3′UTR of PTEN mRNA. Introduction of anti-miR-148a also resulted in depressed Akt signaling by HBx and URG11, resulting in decreased expression of β-catenin. Thus, miR-148a may play a central role in HBx/URG11 mediated HCC, and may be an early diagnostic marker and/or therapeutic target associated with this tumor type.

## Introduction

Chronic hepatitis B virus (HBV) infection is associated with the development of hepatitis, cirrhosis and hepatocellular carcinoma (HCC). HCC is among the five most frequent cancers worldwide [1], [2]. These diseases have few effective treatments [3]. HBV makes a genetic contribution to HCC by expressing the HBV-encoded X antigen, or HBx [4]. HBx is a *trans-*activating protein [5] that contributes to the development of HCC by stimulating cytoplasmic signal transduction pathways [6]–[12] and by acting as a transcriptional co-regulatory protein in the nucleus [13]–[16]. One HBx up-regulated gene, URG11 [17], appears to stimulate hepatocellular growth by transcriptionally activating the β-catenin promoter [18]. This may be a part of the mechanism whereby HBx contributes to HCC [18].

MicroRNAs (miRNAs) are small non-coding RNAs in eukaryotic organisms [19] that play key regulatory roles in mRNA translation and degradation by base pairing to complementary sites in the 3′ un-translated region (3′UTR) of selected transcripts [20]. miRNAs can act as oncogenes or tumor suppressors in carcinogenesis [21]. Aberrant miRNA expression is associated with the pathogenesis of many tumor types, including HCC [21]–[24]. For example, cyclin G1, which is a target of miR122a, is down-regulated in HCC [25]. In this context, it is not known whether HBx or URG11 regulate the expression of miRNAs in hepatocarcinogenesis. When this was explored by miRNA array, several miRNAs were deregulated. Among them, miR-148a was up-regulated by HBx and URG11. Inhibition of up-regulated miR-148a partially blocked the ability of HBx and URG11 to promote tumorigenesis. Further work showed that miR-148a targeted inactivation of the tumor suppressor, phosphatase and tensin homolog (PTEN), which in turn, modulated β-catenin/Wnt signaling. Thus, the ability of HBx and URG11 to stimulate hepatocellular growth via up-regulated expression of β-catenin is modulated by miR-148a, which in turn, is associated with a decrease in PTEN activity.

## Results

### miRNAs Differentially Expressed by HBx or URG11

HepG2, derived from a human hepatoblastoma (a benign tumor), expresses both wild-type and an activated mutant of β-catenin [26]. In contrast, Hep3B, derived from a human hepatoma, encodes only wild-type β-catenin. Hep3B expressing CAT, HBx or over-expressing URG11, were previously used to evaluate β-catenin protein level [18]. Small RNAs isolated from HepG2X and HepG2CAT cultures were subjected to miRNA array analysis. The results showed that 46 miRNAs were differentially expressed (*P* < 0.01). When the same analysis was applied to HepG2URG11 and HepG2CAT cells, 55 miRNAs were differentially expressed (*P* < 0.01) (data not shown). Three miRNAs were up-regulated and five miRNAs were down-regulated in both arrays (Table 1). In this report, miR-148a, which was up-regulated 1.64 -fold in HepG2X and 6.49-fold in HepG2URG11 compared to HepG2CAT cells, was chosen for further characterization.

**Table 1:** Differentially expressed miRNAs in HepG2X and HepG2URG11 compared to HepG2CAT cells by miRNA array.

Designation	Up-regulated	Down-regulated	fold-change^a^	
			HepG2X	HepG2URG1
miR-148a	+		1.64	6.49
miR-146a	+		1.44	2.52
miR-21	+		1.20	1.36
miR-92b		+	1.52	1.26
miR-215		+	1.30	4.33
miR-20b		+	1.32	3.36
miR-27b		+	1.16	1.55
miR-23a		+	1.11	4.46

a fold-changes for each miRNA in HepG2X and HepG2URG11 cells were calculated relative to the corresponding miRNA levels in HepG2CAT cells.

### Confirmation of Up-regulated miR-148a Expression

**Figure 1:**
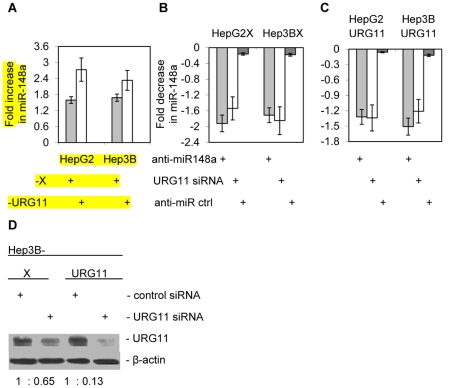
Relationship between HBx, URG11 and miR-148a expression levels. (A) Endogenous levels of miR-148a were determined in the indicated cell lines, and their levels normalized to miR-148a in CAT expressing cells. (B) Cells were transiently transfected with URG11 siRNA (white bars), anti-miR-148a (light gray bars, positive control) or control miRNA (dark gray bars), and the levels of endogenous miR-148a determined. (C) Parallel experiments as in panel A except that HepG2RG11 and Hep3BURG11 cells were used. (D) Relative levels of URG11 protein in cells transiently transfected with URG11 specific siRNA or control siRNA. The results show one of three experiments.

miR-148a expression was quantified in HepG2X, HepG2URG11 and HepG2CAT cells by using SYBR green qRT-PCR. miR-148a was up-regulated 1.59 ± 0.12-fold in HepG2X cells and 2.73 ± 0.46-fold in HepG2URG11 cells compared to HepG2CAT cells (Figure 1A). miR-148a was also up-regulated 1.68 ± 0.11-fold in Hep3BX and by 2.33 ± 0.21-fold in Hep3BURG11 cells compared to Hep3BCAT cells (Figure 1A). Hence, miR-148a was up-regulated in the presence of HBx or over-expressed URG11 in two different liver cell lines.

### Dependence of Elevated miR-148a Upon URG11

To confirm that elevated miR-148a was associated with over-expressed URG11, HepG2 and Hep3B cells expressing HBx or over-expressing URG11 were transiently transfected with siURG11. The results showed that miR-148a levels were depressed by 1.54 ± 0.24-fold in HepG2X cells and depressed by 1.85 ± 0.19-fold in Hep3BX cells (Figure 1B). Parallel experiments using anti-miR-148a for transient transfection (as a positive control) showed that miR-148a levels were down-regulated by 1.92 ± 0.22-fold in HepG2X cells and by 1.71 ± 0.21-fold in Hep3BX cells (Figure 1B). Use of a control siRNA (as a negative control) yielded 0.16 ± 0.02-fold and 0.18 ± 0.018-fold lower levels of miR-148a in HepG2X and Hep3BX cells, respectively (Figure 1B). These results show that up-regulated expression of miR-148a in HBx positive cells is URG11 dependent. This was confirmed in parallel experiments with HepG2URG11 and Hep3BURG11 over-expressing cells (Figure 1C). Control experiments showed that siURG11 suppressed the expression of URG11, demonstrating that this small inhibitory RNA was active (Figure 1D).

### miR-148a Expression in Clinical Specimens

To determine whether HBxAg expression correlated with elevated miR-148a *in vivo*, the expression of HBx and miR-148a was compared in the tumor (T) and nontumor (NT) compartments in 19 patients. HBx staining was strong (intensity 2 or 3) in hepatocytes among 11 of 19 (58%) patients, with mostly lobular or diffuse tissue distribution (Table 2). In contrast, HBx staining was detected in the tumor from only 6 patients (32%), and staining was mostly in scattered cells. Staining was cytoplasmic in both T and NT cells. HBx staining was dominant in NT compared to T in each patient, as shown earlier [4]. Similar staining results were obtained for URG11 (data not shown), as previously published [18]. Additional characteristics of these patients are presented in Table 2.

**Table 2:** Characteristics of HBxAg and miR-148a in tumor and nontumor compartments from HBV-infected HCC patients.

case No.	sex^a^	age	HBsAg (serum)^b^	HBxAg intensity^d^			tissue distribution^e^			qRT-PCR results for miR-148a	
				NT^c^	T^c^	NT^c^		T^c^		ΔΔCt^f^	fold-change^g^
32	1	45	1	1	0	S				−1.14	2.20
39	1	44	1	2	0	L,D				−1.24	2.36
43	1	53	1	1	0	S				+0.63	1.55
86	1	38	1	2	1	L		S		+2.80	6.96
87	1	71	1	2	1	L		S		−4.22	18.64
88	1	52	1	2	1,2	D		S,L		−1.83	3.56
89	1	35	1	1	1.2	S		L		+0.1	1.07
91	1	66	0	1	0	L				−0.7	1.62
95	2	33	1	1,2	1	L		S		+3.11	8.63
98	1	49	1	1	0	S				+0.62	1.54
113	1	58	1	1,2	0	L				−0.74	1.67
114	1	61	1	2	1	D		S		+2.60	6.06
118	1	37	1	2	0	D				+1.02	2.03
140	1	61	1	1	0	D				+6.91	120.26
141	1	57	1	3	0	D				+2.71	6.54
149	1	44	1	1,2	0	D				+1.85	3.61
156	1	50	1	1	0	D				+1.32	2.50
157	1	56	1	3	0	D				+1.45	2.73
202	1	47	1	1	0	L				+4.23	18.77

a. Sex 1  =  male; 2  =  female.

b. Hepatitis b surface antigen (HBsAg) presence in serum: 0  =  no, 1  =  yes.

c. NT  =  non-tumor; T  =  tumor.

d. Intensity of HBxAg staining was evaluated as 0 (no signal) through 3 (intense signal).

e. Tissue distribution of HBxAg: S  =  scattered, L  =  lobular, D  =  diffuse.

f. Negative ΔΔCt value  =  elevated expression of miR-148a in tumor; positive ΔΔCt value  =  elevated expression of miR-148a in non-tumor liver.

g. Fold change is calculated as 2^ΔΔCt^. The average fold change for elevated miR-148a was calculated by adding up the fold-change from each patient with a positive ΔΔCt value divided by the number of patients with a positive ΔΔCt value. The positive ΔΔCt values listed in this table are plotted in Figure 1 and represent elevated miR-148a levels in liver compared to tumor. Parallel calculations were performed to determine the average fold change among patients with negative ΔΔCt values, which are plotted in Figure 1 as elevated miR-148a levels in tumor compared to liver.

**Figure 2:**
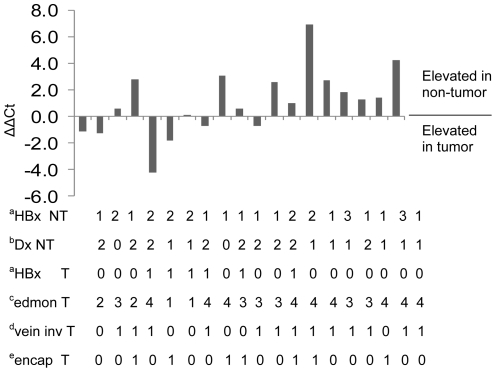
Expression of miR-148a in tumor and non-tumor liver tissues. Each bar represents data collected from one patient. The difference in miRNA expression between tumor (T) and non-tumor (NT) was determined by qRT-PCR and determination of ΔΔCT, where ΔΔCt  =  ΔCt of miR-148a in tumor – ΔCt of miR-148a in non-tumor. U6 was used for normalization. Negative values indicate that miR-148a levels were higher in tumor compared to adjacent non-tumor. Positive values indicate that miR-148a levels were higher in NT (liver) compared to T. ^a^HBx NT, HBx T: HBx staining in tumor (T) and nontumor (NT) is scored as follows: 0  =  negative, 1  =  up to 25% of cells stained positive, 2  =  25–50% of cells stained positive, 3  =  > 50% cells stained positive. ^b^Dx NT refers to diagnosis of lesions in nontumor liver. These are as follows: 0  =  no significant lesions, 1  =  chronic hepatitis, 2  =  cirrhosis. ^c^edmon T refers to the Edmondson classification of cellular differentiation within tumor nodules. They are as follows: 1  =  Edmondson I-II, 2  =  Edmondson II, 3  =  Edmondson II-III, 4  =  Edmondson III. dVein inv T refers to venous invasion of the tumor nodule, where 0  =  no evidence for invasion, and 1  =  presence of venous invasion. eEncap T refers to tumor encapsulation, where 0  =  none and 1  =  encapsulation.

Total small RNA was extracted separately from T and NT tissues from these same patients. The expression of miR-148a was then determined by SYBR Green qRT-PCR. The ΔΔCt values showed that miR-148a was elevated in 13 out of the 19 NT samples (68%) and in 6 out of 19 tumors (31%) (Table 2). This corresponds to an average of 14-fold change in miR-148a levels in NT in 13 patients and an average of 5-fold change in T from the remaining 6 patients (Table 2). HBx expression in NT was associated with chronic hepatitis (*P* < 0.02), cirrhosis (*P* < 0.01) and elevated levels of miR-148a (*P* < 0.001) compared to uninfected liver. Thus, HBx is associated with up-regulated expression of miR-148a in NT compared to T by an average of (14 ÷5) 2.8-fold. This is similar to results seen with HepG2X and HepG2URG11 compared to control cells. Thus, elevated miR-148a expression appears to be an early event in the pathogenesis of HCC, since it was observed most often in infected liver tissues from which tumor nodules developed. Further, elevated miR-148a in NT was associated with Edmond III-IV stage tumor (*P* < 0.001) and venous invasion (*P* < 0.001) but not with a tumor capsule (*P* > 0.25). These observations suggest that elevated miR-148a triggered changes in host gene expression that resulted in the appearance of more aggressive tumors despite the fact that miR-148a expression was not elevated in most tumors (Figure 2, Table 2).

### Anti-miR-148a Inhibits Cell Growth and Viability

**Figure 3:**
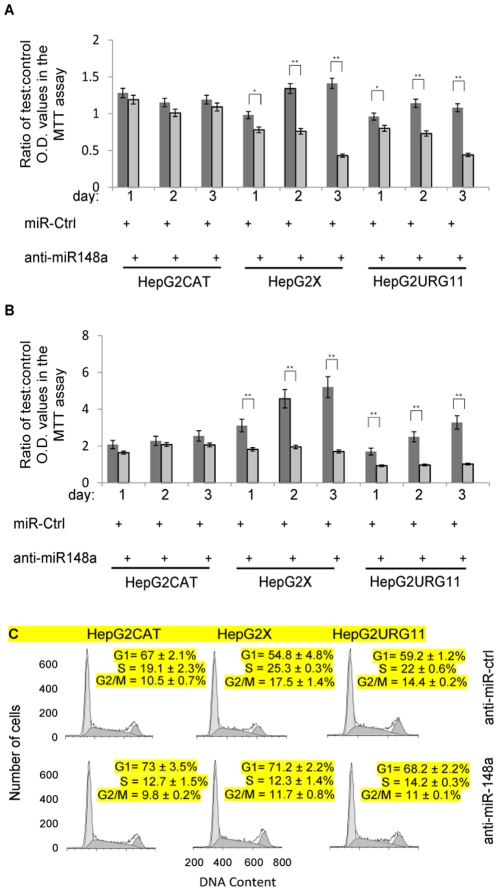
Effect of anti-miR-148a on cell phenotype. HepG2CAT, HepG2X and HepG2URG11 cells were (A) transiently transfected with anti-miR-148a or (B) stably transduced with recombinant lentivirus encoding anti-miR-148a. In both cases, cell growth was evaluated by MTT assay on days 1, 2 and 3 of culture. All values were normalized to cells treated only with transfection reagent and are presented as a ratio of test:control O.D. values. (C) Serum starved cells were analyzed by flow cytometry on day 3 after the addition of serum. The percentage of cells in G1,S,G2/M phases are indicated in each panel.

To test whether HBx and URG11 stimulated cell growth is at least partially dependent upon miR-148a, HepG2X and HepG2URG11 cells were transiently transfected with anti-miR-148a. The results showed that anti-miR-148a significantly inhibited cell growth on all days post-transfection, and by day 3, inhibition was 60–70% (Figure 3A). Neither control miRNA introduced into HepG2X or HepG2URG11 cells, nor introduction of anti-miR-148a into HepG2CAT cells, inhibited growth at any point in time. However, significant growth inhibition was observed in Hep3BX and Hep3BURG11 compared to Hep3BCAT cells (data not shown). Transfection efficiency was monitored with a Cy5-labled-miRNA under the same experimental conditions and was estimated to be close to 100% (data not shown). These observations suggest that HBx and URG11 promote cell growth, in part, by up-regulated expression of miR-148a.

To confirm and extend the functional characterization of miR-148a, HepG2 and Hep3B cells encoding HBx, URG11 or CAT were stably transduced with recombinant lentivirus encoding anti-miR-148a. Growth of HepG2X cells stably expressing anti-miR-148a was inhibited by an average of 68% by day 3 (*P* < 0.01). For HepG2URG11, anti-miR-148a inhibited growth an average of 69% by day 3 (*P* < 0.01) (Figure 3B). Similar inhibition was observed in Hep3BX and Hep3BURG11 cells stably expressing anti-miR-148a compared to control miRNA (data not shown). Growth of HepG2CAT cells was not altered by anti-miR-148a. These findings again suggest that HBx and URG11 stimulate cell growth, at least in part, in a miR-148a dependent manner.

To see if elevated miR-148a also promotes cell cycle progression, Hep3BCAT, Hep3BX, Hep3BURG11 stably expressing anti-miR-148a or control anti-miR were synchronized by serum starvation, released by addition of serum, and then subjected to flow cytometry. Day 3 results show that anti-miR-148a suppressed cell cycle progression into S phase in Hep3BX (*P* < 0.005) (Figure 3C). Similar results were seen in Hep3BURG11 (*P* < 0.01), and to a lesser extent in Hep3BCAT cells (*P* < 0.05). The same trends were observed in these cells at the G2/M transition, suggesting that miR-148a promotes cell cycle progression, especially in URG11 over-expressing and HBx expressing cells.

### Anti-miR-148a Inhibits Cell Migration

**Figure 4:**
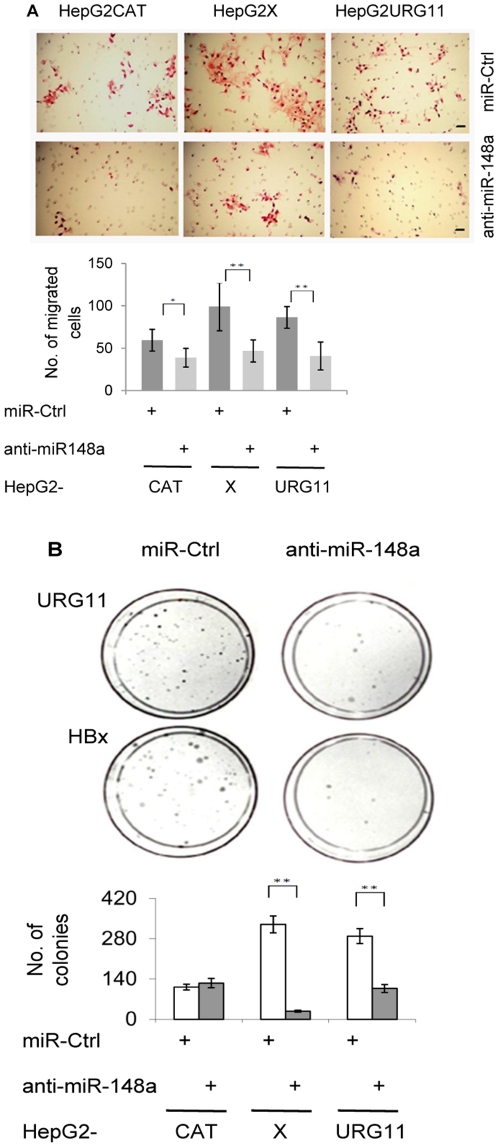
Effect of anti-miR148a on cell migration and anchorage independent growth. (A) Migration of HepG2CAT, HepG2X, and HepG2URG11 cells stably expressing anti-miR-148a or control anti-miR. The top portion of this panel shows the results of a single experiment, while the graphical representation below is the average from 3 independent experiments. The black bar in the photomicrographs is 50um. (B) Summary of soft agar assays for HepG2CAT, HepG2X and HepG2URG11 cells stably expressing anti-miR-148a or control anti-miR. The average number of colonies reported here was from three independent experiments performed in triplicate. *  =  *P* < 0.05; **  =  *P* < 0.01 in the Student’s t test.

Increased cell migration is another characteristic of tumor cells. Thus, migration of HepG2CAT, HepG2X and HepG2URG11 cells were evaluated with or without anti-miR-148a. The results showed that anti-miR-148a, but not control anti-miR, partially blocked the ability of HBx and URG11 to promote migration of HepG2 cells after 72 hr (*P* < 0.01) (Figure 4A). In this case, HepG2CAT cell migration was also modestly inhibited (*P* < 0.05), suggesting that miR-148a promotes cell migration to a greater extent in HBx or over-expressed URG11 cells compared to control.

### Anti-miR-148a Blocks Colony Formation in Soft Agar and Tumor Formation in SCID Mice

Prior work showed that HBx and URG11 promoted growth in soft agar and tumor formation in nude mice [17]. To test whether miR-148a contributed to tumorigenesis, HepG2CAT, HepG2X, HepG2URG11 cells stably expressing anti-miR-148a or control anti-miR were evaluated for anchorage independent growth in soft agar. Anti-miR-148a suppressed colony formation of HepG2X cells by an average of 11.8-fold, and that of HepG2URG11 cells by an average of 2.7-fold, compared to control anti-miR (Figure 4B, *P* < 0.01). In contrast, anti-miR-148a did not suppress HepG2CAT cell growth in soft agar compared to control miRNA treatment.

The impact of miR-148a upon tumorigenicity was then assessed by xenotransplantation. For HepG2X and HepG2URG11 cells expressing anti-miR-148a, tumor growth was partially inhibited (Table 3), suggesting that up-regulated miR-148a contributes to HBx and URG11 mediated tumor growth. In contrast, inhibition of miR-148a in HepG2CAT cells had little impact upon tumorigenicity. These observations suggest that miR-148a, at least in part, drives tumor growth mediated by HBx, and in particular, by over-expression of URG11.

**Table 3:** Role of miR-148a in tumorigenesis.

Cell line	Treatment	average weight of tumors (gms)	student’s t test (*p*-value)
HepG2X	anti-miR-Ctrl anti-miR-148a	1.48 ± 0.72 0.90 ± 0.44	< 0.05
HepG2URG11	anti-miR-Ctrl anti-miR-148a	1.30 ± 0.60 0.38 ± 0.43	< 0.04
HepG2CAT	anti-miR-Ctrl anti-miR-148a	0.40 ± 0.33 0.34 ± 0.25	> 0.5

### miR-148a Targets PTEN

**Figure 5:**
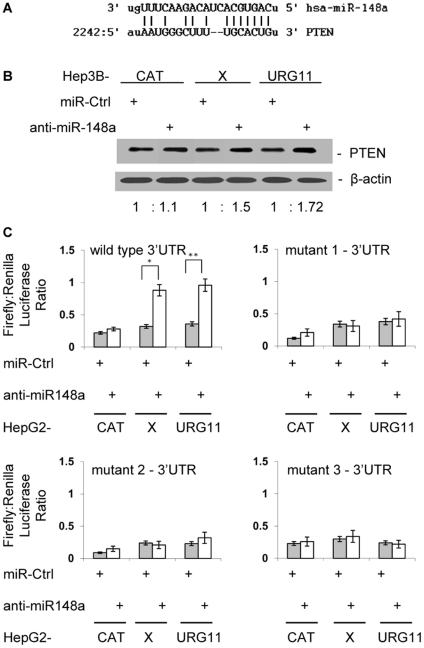
Effect of anti-miR-148a on PTEN expression. (A) Homology between the PTEN 3′UTR and anti-miR-148a. (B) PTEN protein levels in cells stably expressing anti-miR-148a or anti-miR control were determined by western blotting. (C) HepG2CAT, HepG2X, HepG2URG11 cells were transiently co-transfected with anti-miR-148a or anti-miR-Ctrl and a PTEN 3′UTR expression plasmid. Firefly (test) and Renilla (control) luciferase activities were measured after 48hr. The ratios were calculated from the Firefly luciferase: Renilla luciferase readings. *  =  *P* < 0.05 and **  =  *P* < 0.01 in the Student’s t test.

Putative human gene targets of miR-148a were identified by using miRanda, TargetScan, and PicTar algorithms. One of the target genes is the tumor suppressor, PTEN. PTEN expression is down-regulated by HBx [27]. The homology between miR-148a and PTEN from miRanda is shown in Figure 5A. In HepG2CAT, HepG2X, HepG2URG11 cells transiently transfected with anti-miR-148a, PTEN mRNA levels increased 1.19 ± 0.22-fold in HepG2CAT cells, 1.25 ± 0.17-fold in HepG2X cells and 1.34 ± 0.20-fold in HepG2URG11 cells compared to cells transiently transfected with control anti-miR. At the protein level, total PTEN increased 1.5 ± 0.15-fold in HepG2X and 1.72 ± 0.18-fold in HepG2URG11 cells compared to 1.1 ± 0.1-fold in HepG2CAT cells (*P* < 0.01) (Figure 5B). To test whether the predicted miR-148a target site in the 3′UTR of PTEN mRNA was responsible for its regulation, the 3′UTR target site downstream from a luciferase reporter gene (pEZX-PTEN-3′UTR) was co-transfected with either anti-miR-148a or anti-miR control. HepG2X cells transiently transfected with anti-miR-148a had significantly higher luciferase activity (2.66-fold, *P* < 0.02), and so did HepG2URG11 cells (2.75-fold, *P* < 0.01), compared to cells treated with control anti-miR (1.27-fold) (Figure 5C). Given that the PTEN 3′UTR contains two miR-148a binding sites, the wild type pEZX-PTEN-3′UTR was mutated at each or both of these sites (Table S1). Parallel experiments using reporter plasmids containing these mutations resulted in little increase in luciferase activity, suggesting that the mutant PTEN 3′UTRs did not bind to miR-148a (Figure 5C). Taken together, these data suggest that the binding of miR-148a to the 3′UTR of PTEN is specific, and that PTEN is a target of miR-148a.

### miR-148a and Akt Signaling

**Figure 6:**
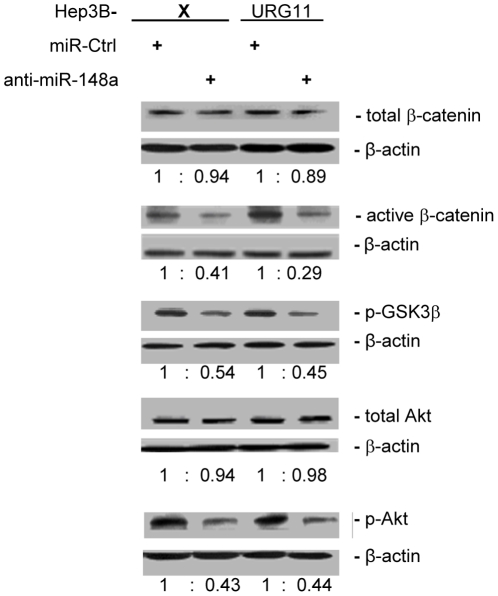
Effect of anti-miR-148a upon the expression levels of Akt, GSK3β, and β-catenin. The indicated cell lines were stably transfected with anti-miR-148a (or control miRNA) and then analyzed for total and active β-catenin, p-GSK3β, as well as total and p-Akt by western blotting. The relative amounts of each, normalized to β-actin, are presented below each blot.

Since PTEN blocks PI3K activity, experiments were designed to test whether anti-miR-148a blocks Akt signaling by activating PTEN and thereby suppressing β-catenin expression. To test this hypothesis, total and active β-catenin, phosphorylated-GSK3β (p-GSK3β, Ser9), as well as total and phosphorylated-Akt (p-Akt, Ser473) levels, were determined in Hep3BX and Hep3BURG11 cells stably expressing anti-miR-148a. Stably expressed anti-miR-148a was associated with essentially stable levels of total Akt but significantly decreased levels of activated Akt (p-Akt; *P* < 0.005). Likewise, total levels of β-catenin were minimally altered by stable expression of anti-miR-148a, while the levels of active β-catenin were depressed in Hep3BX cells (*P* < 0.05), and in Hep3BURG11 cells (*P* < 0.01), compared to controls (Figure 6). Further, the inactive form of GSK3β, p-GSK3β, was depressed by treatment with anti-miR-148a (*P* < 0.05). These results suggest that anti-miR-148a inhibits Akt signaling, which results in lower levels of active β-catenin. qRT-PCR analysis of Akt, GSK3β, and β-catenin mRNAs showed no differences in cells treated with anti-miR-148a or control anti-miR (data not shown). Thus, up-regulated miR-148a, through PTEN, may impact upon Akt signaling post-translationally.

### Relationship between URG11 and PTEN

**Figure 7:**
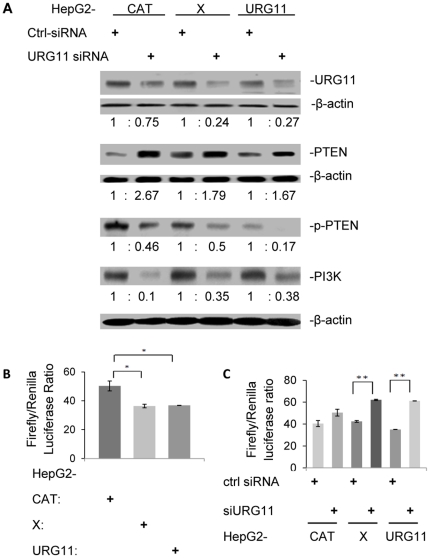
The relationship between URG11 and PTEN. (A) URG11, PTEN, p-PTEN and PI3K protein expression in the indicated cultures transiently transfected with siURG11 or control siRNA. The numbers listed under each blot represent the band intensities after normalization to β-actin. (B) PTEN promoter activity (indicated by firefly luciferase activity) and phRL_null (the renilla luciferase control) were transiently co-transfected into the indicated cell lines. Luciferase activities were measured 48 hrs later. (C) PTEN promoter activity was assayed in cells 48 hrs after transient transfection with siURG11 or control siRNA. Each of the experiments in this figure was repeated at least three times.

Since over-expression of URG11 directly correlates with up-regulated expression of miR-148a (Figure 6), and miR-148a depresses PTEN protein expression (Figure 5), URG11 may activate β-catenin by suppressing PTEN. The latter would result in the activation of PI3K/Akt signaling [28], [29]. To test this, siURG11 was transiently transfected into HepG2CAT, HepG2X, HepG2URG11 cells and the protein levels of PTEN, phosphorylated (inactivated) PTEN (p-PTEN, Ser380/Thr382/383) and PI3K were determined. The results showed that siURG11 partially suppressed URG11 protein levels (Figure 7A) (*P* < 0.01), indicating siURG11 was functional. siURG11 treatment increased levels of total PTEN (*P* < 0.02), and depressed the levels of p-PTEN (*P* < 0.01). Treatment with siURG11 also resulted in decreased PI3K levels (Figure 7A) (*P* < 0.02). Together, these results suggest URG11 activates PI3K by suppressing PTEN. This was supported by the results of qRT-PCR, which showed that siURG11 treatment up-regulated PTEN mRNA 2.1 ± 0.14-fold in HepG2CAT cells, 1.73 ± 0.22-fold in HepG2X cells and 3.0 ± 0.35-fold in HepG2URG11 cells (data not shown). When these cultures were treated with PTEN siRNA (siPTEN), URG11 protein and mRNA levels were unchanged (data not shown). Hence, PTEN does not have any impact upon URG11 expression. However, siPTEN enhanced Akt and β-catenin expression (data not shown), as previously published [30] **.**

The finding that URG11 inhibits PTEN raises the possibility this inhibition may also occur at the PTEN promoter. To test this, PTEN promoter activity was determined in HepG2CAT, HepG2X, HepG2URG11 cells. In URG11 over-expressing or HBx expressing cells, PTEN promoter activity was decreased 28% compared to HepG2CAT cells (*P* < 0.05) (Figure 7B). When this experiment was repeated by transiently transfecting siURG11, there was a significant increase in PTEN promoter activity in HepG2X and HepG2URG11 cells compared to parallel cultures treated with control siRNA (*P* < 0.01) (Figure 7C). However, immunoprecipitation (IP) failed to show any binding between PTEN and URG11 (data not shown). Thus, URG11 may also inhibit PTEN through suppressing the PTEN promoter.

## Discussion

**Figure 8:**
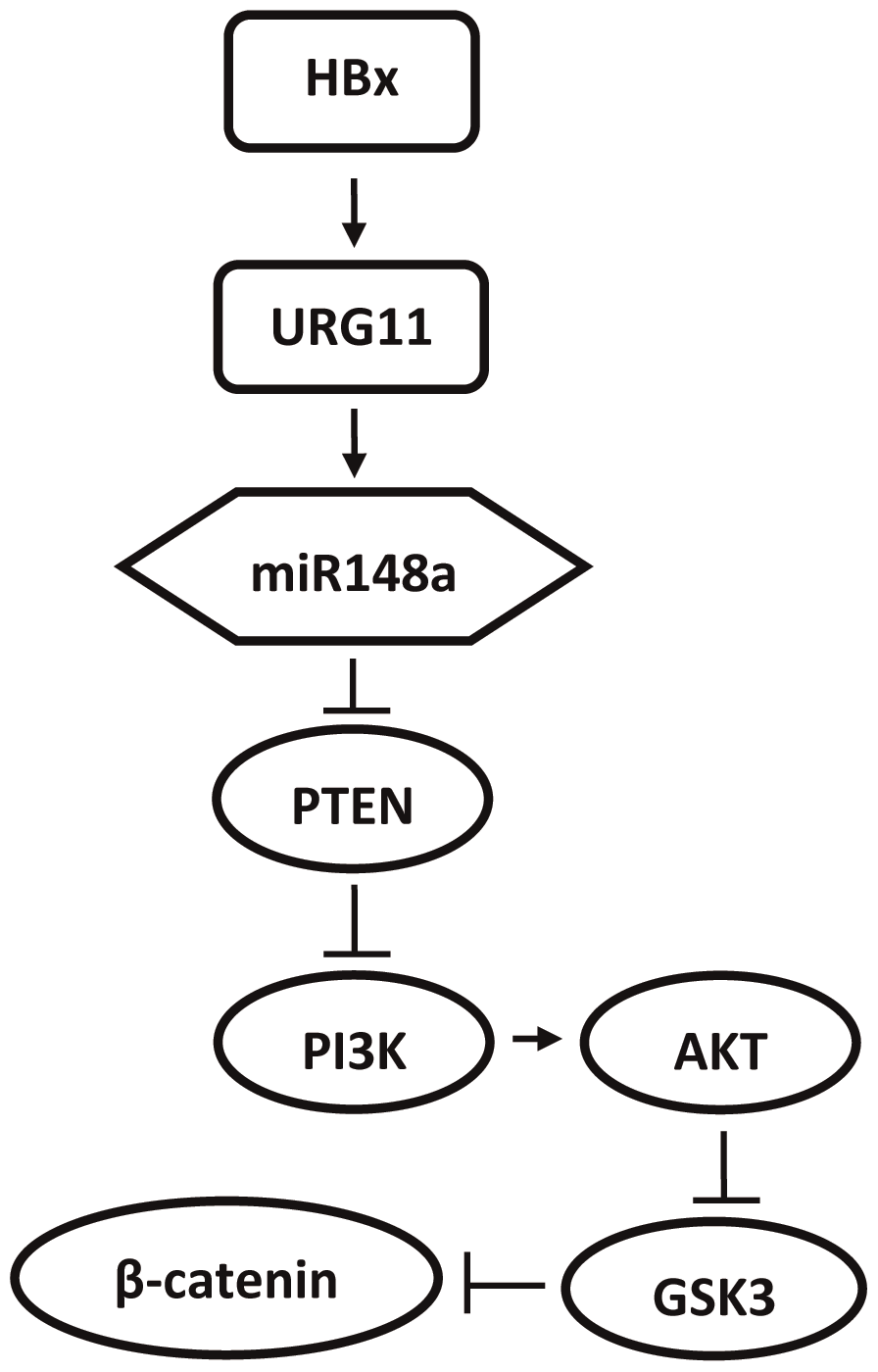
Model of miR148a associated signaling pathway. miR148a increases β-catenin expression by inhibiting PTEN. See the text for additional details.

Deregulated expression of miRNAs has been reported in many human malignancies [31]–[33]. Functional characterization of these miRNAs and their target proteins in tumorigenesis has been important in identifying novel therapeutic targets [31], [34]–[36]. Given the centrality of HBx to HBV associated HCC [4], and that the HBx target, URG11 strongly stimulates hepatocellular growth and tumorigenesis [18], miRNA array analysis was conducted with HepG2X, HepG2URG11 and HepG2CAT cells to identify differentially expressed miRNAs. The results (Table 1) identified miR-148a as one of the up-regulated miRNAs in cells expressing HBx or over-expressing URG11. In 19 T/NT tissue pairs from as many patients with HBV associated HCC, miR-148a was up-regulated an average of 14-fold in NT tissue from 13 patients. In the remaining 6 patients, miR-148a was elevated an average of 5-fold in T (Table 2, Figure 2). miR-148a stimulated cell viability, cell migration, anchorage independent cell growth and tumorigenesis in SCID mice (Table 3, Figure 3–4) and appears to target the tumor suppressor PTEN (Figure 5). Inactivation of PTEN resulted in the activation of PI3K/Akt and β-catenin (Figure 6) and may involve URG11 inhibition of the PTEN promoter (Figure 7). Thus, HBx up-regulation of URG11 and miR-148a may be two mechanisms that block PTEN activity, resulting in the activation of β-catenin signaling (Figure 8). This supports earlier work showing that HBx stimulated PI3K/Akt and stabilized β-catenin [37].

URG11 was discovered when HepG2X and HepG2CAT cells were subjected to subtractive hybridization [17]. The location of up-regulated URG11 in hepatocytes surrounding tumor nodules, and that it stimulates cell growth by activating wild type β-catenin, suggests that this protein promotes early stages of HCC [18]. The finding herein, that URG11 over-expression is associated with elevated expression of miR-148a, which then blocks the translation of PTEN, contributes importantly to understanding the centrality of URG11 in the activation of PI3K/Akt and β-catenin. The fact that the tumor suppressor, p53, activates expression of PTEN [38], and that HBx inactivates p53 and PTEN [27], [39]–[41] provides another mechanism whereby PTEN inactivation contributes to HCC. The ability of PTEN to up-regulate p21^WAF1/CIP1/SDI1^ [42], and that HBx suppresses p21^WAF1^ expression [43], suggest that the HBx inactivation of PTEN accelerates cell cycle progression, which was seen herein. Inactivation of PTEN also correlates with activation of PI3K/Akt, resulting in the up-regulation of MDM-2, which promotes tumorigenesis [44]. The fact that PTEN is absent in about 50% of HCC cases [45] suggest that loss of this tumor suppressor is common. Further, the finding that HBx constitutively activates oncogene signaling in the liver may be a mechanism whereby HBV may overcome oncogene induced senescence [46].

miR-148a was first shown to block apoptosis [47] by modulating the levels of cytochrome P450 3A4 via post-transcriptionally regulating the 3′UTR of the Pregnane X Recepter (PXR) mRNA [48]. Since PXR contributes to the detoxification of xenobiotics in the liver [49]–[51], the inverse relationship between miR-148a and PXR in chronic liver disease (CLD) may promote toxic liver damage. The function of miR-148a is also likely to be cell type dependent, since it is down-regulated in acute myeloid leukemia [52], [53]. In addition, down-regulated expression of miR-148a by hypermethylation was associated with metastasis in many tumor types [54], and with up-regulation of metastasis associated genes such as subunit 1 of the general transcription factor IIH [55]. miR-148a was also shown to repress DNA methyltransferase 1 (DNMT1) and DNMT3B [56], [57]. HBx activates DNMT1, which suppresses the tumor suppressor, p16^INK4a^ [58]. Importantly, most HBx and up-regulated miR-148a were found in the NT compartment of clinical samples from tumor bearing patients (Figure 2, Table 2), suggesting that epigenetic changes involving hypermethylation occur prior to tumor appearance and may promote carcinogenesis. In the present work, up-regulated miR-148a in the liver was associated with the appearance of aggressive tumors (Edmonson grade III-IV) characterized by venous invasion (Figure 2, Table 2) and by the ability of elevated miR-148a to promote tumorigenesis (Table 3). However, the fact that up-regulated miR-148a was found in mostly NT herein, combined with its ability to stimulate viability, proliferation, migration and growth in soft agar (Figure 3–4), suggest that tissue remodeling and metastasis are early events in HBV associated HCC instead of late events associated with tumor progression. Since CLD involves tissue regeneration and remodeling, up-regulation of miR-148a in the chronically infected liver may promote the transition of hepatocytes into tumor cells. Once accomplished, miR-148a (and HBx) is no longer selected for, and their expression levels decrease. Thus, miR-148a may promote tumorigenesis from the non-tumor compartment but is no longer active once tumor appears.

PTEN is suppressed by miR-148a in HBV associated HCC (Figure 5) although other mechanisms may also be operative. For example, PTEN is down-regulated by miR-214 in ovarian cancer [59]. In addition, miR-21 which down-regulates PTEN, is up-regulated in liver and HCC [60], suggesting up-regulated miR-21 is associated with disease progression. The finding of elevated miR-21 in HepG2X and HepG2URG11 compared to HepG2CAT cells, suggests that it may also be a target of HBx. Further, miR-216a and miR-217, both of which target PTEN, activate Akt through PTEN down-regulation in kidney disorders [61]. Additional work will be needed to investigate whether miR-148a targets other proteins in addition to PTEN that are important to pathogenesis of HBV-mediated HCC. Antagonists of endogenous miR-148a may be a useful therapeutic strategy for enhancing PTEN expression and suppressing Akt levels in the HBV infected liver.

## Materials and Methods

### Cell Lines

HepG2 and Hep3B cells constitutively expressing CAT, HBx, or URG11 were constructed in the lab and used as previously reported [18]. Hep3BCAT, Hep3BX, Hep3BURG11 were constructed in the same way as previously reported [18]. All were made by transduction using recombinant retroviruses encoding CAT, HBx or URG11. Each transduced culture was treated with G418 (800 ug/ml; GIBCO/BRL, Grand Island, NY) for 14 days to remove uninfected cells, and each culture propagated without selection of individual colonies. For the detection of CAT activity, equivalent amounts of cell lysates were incubated with ^14^C-chloramphenicol, and the extent of acetylation analyzed by thin layer chromatograpy and autoradiography. HBx and URG11 expressing cultures were initially characterized by western blotting for these recombinant proteins. Cultures were then propagated in G418 indefinitely. Additional details of these cultures were previously published [8].

### Small RNA Isolation

Small RNAs were isolated from cells and 19 pairs of HCC tissue samples using the mirVana miRNA isolation kit and PARIS miRNA isolation kit (both from Ambion, Austin, TX), respectively, by the enclosed protocols. The concentration of small RNA was determined by O.D. 260/280nm absorbance.

### Identification of HBx-related miRNA by Microarray Analysis

RNA samples from HepG2X and HepG2CAT cells, 50 µg from each cell line, were sent to LC Sciences (Houston, TX) for paired miRNA microarray analysis. Differentially expressed HBx-related miRNAs were identified by Cy3 (HepG2X)/Cy5 (HepG2CAT) ratio and *P* < 0.01. RNA samples from HepG2URG11 and HepG2CAT cells were analyzed by the same protocol. The results reported herein represent the average fold change in expression of each miRNA from three independently performed experiments in Table 1. Both arrays were compliant to MIAME guidelines and deposited in GEO with accession number GSE33854.

### Patient Tissues

Nineteen pairs of fresh and paraffin-embedded samples, each containing tumor (HCC) and adjacent non-tumor (liver), were collected from HCC patients treated at Queen Mary Hospital of Hong Kong University. These samples had accompanying documentation for clinical, pathological and laboratory evaluations in Table 2. These samples were de-identified and their use was approved by the institutional review boards from both Hong Kong University and Temple University.

### Quantification of miRNA Expression in Cells and Tissues by qRT-PCR

Tissue and cell line miRNA expression was determined by real time quantitative RT-PCR using Eppendorf’s realplex Mastercycler following the manufacturer’s protocol. Briefly, 25 ng of small RNA was used for each qRT-PCR reaction. The PCR reaction mixture was denatured at 95°C for 15 min and then run for 40 cycles (94°C for 15 sec, 55°C for 30 sec, and 70°C for 30 sec). Melting curve analysis was run at the same time to rule out non-specific reactions or contamination. U6 was used for normalization. Agarose gel electrophoresis, using 2.5% gels, was used for verification of uncertain results. All qRT-PCR were run in triplicates. The difference in miRNA expression was determined by ΔΔCT. mirVana qRT-PCR miRNA Detection Kit, reverse transcriptase and PCR primer sets (hsa-miR-148a and U6), miR-negative control 1, and hsa-miR-148a inhibitor (anti-miR-148a) were all purchased from Ambion.

### URG11 Specific Small Interfering RNAs (siURG11) and Primers

The PTEN siRNA kit was purchased from Cell Signaling Technology (Danvers, MA). URG11 siRNA sequence and protocol to transfect cells were described previously [18]. Parallel wells were transfected with non-targeting siControl #1 siRNA or with transfection reagent only (DharmaFECT1 from Dharmacon, Lafayette, CO). The primers designed for URG11 and PTEN mRNA are: URG11 forward: tgtgccgagactgcaactac; URG11 reverse: gcaatctggacaggaagagc; PTEN forward: cgacgggaagacaagttcat; PTEN reverse: gctag cctctggatttgacg; β-actin forward: aagagctatgagctgccga; β-actin reverse: tacggatgtcaacgtcacac.

### Establishment of pmiRZip-148a Stable Expressing Cell Lines

Three million 293TN cells in a fresh 10-cm plate were plated and transfected with 10 µg of the pPACK Packaging Plasmid mixed with 2 µg of pmiRZip-148a or corresponding control vector using LipofectamineTM (Invitrogen, Carlsbad, CA). Supernatants were collected at 48 hrs post-transfection, clarified by centrifugation, mixed with Polybrene, and then used for transduction. After 72 hrs, cells were selected with 100ug/ml puromycin for 2 weeks, and then the lentivirus infected cultures enriched by fluorescence activated cell sorting, and propagated long term. Lentiviral based anti-mir-148a construct (pmiRZip-148a) and control vector (pGreenPuro Scramble Hairpin Control), pPACKF1™ Lentivector Packaging Kit and the 293TN producer cell line were all purchased from System Biosciences (Mountain View, CA).

### Effects of miRNAs on Cell Growth

Cells were seeded in triplicate on collagen coated 96 well plates (5,000 cells/well) (Nunc, Rochester, NY) and cultured in complete medium over night at 37°C and 5% CO2. Cells were then transiently transfected with 100 ng of anti-miR-148a (Ambion) using DharmaFECT1. Cell proliferation was determined on days 1, 2 and 3 in the CellTiter 96 AQueous One Solution Cell Proliferation Assay (Promega, Madison, WI). miR-negative control 1 was used for normalization.

### Anchorage Independent Growth, Tumorigenesis and Cell Migration Assays

To evaluate anchorage independent growth, single cell suspensions of 1 x 10^4^ cells were mixed with 0.4% agar (Sigma, St. Louis, MO) in complete growth medium and seeded in triplicates into 6-well plates coated with 0.8% hardened agar. Plates were incubated at 37°C for 28 days. Colonies ≥ 1 mm in diameter were counted under code by light microscopy.

For tumorigenicity studies, two groups of 10 six-week-old SCID mice (Charles River Laboratories, Wilmington, MA) were injected subcutaneously at a single site with 6 x 10^6^ cells. Tumor onset was scored visually and by palpitation independently by two trained lab personnel. Tumor sizes were determined by wet weight at the time of euthanasia (6 weeks). These experiments were approved by the Institutional Animal Care and Use Committee at Temple University.

To evaluate cell migration, single cell suspensions of 1.5×10^5^ cells were plated in triplicates into 6-well BD BioCoat™ Matrigel™ Invasion Chambers (BD, Franklin Lakes, NJ) according to enclosed instructions. Cell migration was observed after 24h by hematoxylin and eosin (H & E) staining.

### PTEN 3′UTR Constructs and luCiferase Reporter Assay

The pEZX-PTEN-3′UTR construct (Genecopoeia, Rockville, MD), which contains the PTEN 3′UTR sequence, has the putative binding site for miR-148a downstream of the firefly luciferase stop codon. It also contains the Renilla luciferase sequence downstream of the early CMV promoter as an internal control. Three additional constructs, identical to pEZX-PTEN-3′UTR but each containing unique point mutations in one or both miR-148a binding sites, were also made (Genecopoeia) and tested. The sequences of these mutants are listed in Table S1. Cells were plated (5,000 cells/well) in 96 well plates and co-transfected with 100ng of the pEZX-PTEN-3′UTR and 100 ng of anti-miR-148a, using DharmaFECT1. Luciferase assays were performed after 48 hrs using the Luc-Pair miR Luciferase Assay (Genecopoeia) in a Glowmax luminometer (Promega, Madison, WI). Firefly luciferase activity was normalized to renilla luciferase expression for each sample.

### PTEN Promoter Luciferase Assay

The PTEN promoter luciferase plasmid was purchased from Switchgear Genomics (Menlo Park, CA). The phRL-Null Renilla luciferase plasmid (Promega) was used as an internal control. Co-transfection of 100 ng of PTEN promoter luciferase and 10 ng of phRL-Null was performed with DharmaFECT1. Luciferase activity was determined by the Promega Steady-Glo Luciferase Assay Reagent (Promega). To determine whether URG11 inhibits PTEN promoter activity, URG11 specific siRNA was co-transfected with 100 ng of PTEN promoter luciferase and 10 ng of phRL-Null. Parallel wells were transfected with non-targeting siControl #1 siRNA.

### Western Blotting

Sodium dodecyl-sulfate-polyacrylamide gel electrophoresis and western blotting were carried out as described earlier [18]. Samples containing 30–50 µg of protein were prepared in RIPA buffer for analysis. Primary antibodies included rabbit anti-URG11 [17] (1:2000), rabbit anti-PTEN (1:1000), rabbit anti-phospho-PTEN (Ser380/Thr382/383, 1:1000) rabbit anti-phospho-Akt (Ser 473, 1:1000), rabbit anti-Akt (1:1000), rabbit anti-GSK3β (Ser9, 1:1000) and rabbit anti-PI3K (D4E2, 1:1000) (all from Cell Signaling Technology), mouse monoclonal antibody against activated wild-type β-catenin (clone8E7,1:500, Millipore, Burlington, MA), and mouse monoclonal β-catenin antibody (E-5, 1:1000, Santa Cruz Biotechnology, Santa Cruz, CA). HRP labeled second antibodies against mouse (1:5000) and rabbit (1:5000) IgG were purchased from Sigma Chemical Co. (St. Louis, MO). Mouse anti-β-actin monoclonal antibody (clone AC-15, Sigma, 1:10,000) was used as an internal control for normalization. All signals were detected by Amersham ECL PlexTM (Piscataway, NJ).

### Statistics

Differences in miRNA expression between tumor and non-tumor were arbitrary determined by ΔΔCT. The Student’s t test was used to analyze cell growth. The relationships between HBx, miR-148a, and histopathology were assessed by 2×2 Chi-square analysis. In both tests, a significant relationship was indicated when *P* < 0.05. *  =  *P* < 0.05. **  =  *P* < 0.01.

## Supporting Information

Table S1 **Sequence of PTEN 3′UTR expression plasmids.**
(DOCX)
