# Supplementary material for: Role of miR-148a in Hepatitis B Associated Hepatocellular Carcinoma
Source: PLoS One. 2012 Apr 9;7(4):e35331. doi: 10.1371/journal.pone.0035331 (PMC3322146; doi:10.1371/journal.pone.0035331)
Supplement: Table S1 — Sequence of PTEN 3′UTR expression plasmids. (DOCX) [file pone.0035331.s001.docx]

Table S1. Sequence of PTEN 3’UTR expression plasmids

| Name | Type | Nucleotide residues | Sequence* |
| --- | --- | --- | --- |
| hsa-miR-148a | wt |  | 3' UGUUUCAAGACAUCACGUGACU 5' |
| PTEN 3’UTR | wt | 2254-2260 | 5'  AUUAUAAUGGGCUUUUGCACUGU 3' |
| PTEN 3’UTR | mt1 | 2254-2260 | 5’ AUUAUAAUGGGCUUUUGCAgacU 3’ |
| hsa-miR-148a | wt |  | 3’ UGUUUCAAGACAUCACGUGACU 5’ |
| PTEN 3’UTR | wt | 3151-3157 | 5' UGUGUGUAAAUGCUAUGCACUGA 3’ |
| PTEN 3’UTR | mt2 | 3151-3157 | 5' UGUGUGUAAAUGCUAUGCAgacA 3’ |
| PTEN 3’UTR | mt3 | 2254-2260, 3151-3157 | mutations in mt1 plus mt2 |

*Underlined regions of the PTEN 3’UTR are binding sites for miR-148a. mt = mutant; wt = wild type.
